# Patch Angioplasty or Primary Closure Following Carotid Endarterectomy for Symptomatic Carotid Artery Stenosis

**DOI:** 10.1055/s-0038-1655757

**Published:** 2018-06-15

**Authors:** Eline Huizing, Cornelis G. Vos, Robin G. Hulsebos, Peter J. van den Akker, Gert Jan de Borst, Çağdaş Ünlü

**Affiliations:** 1 Department of Surgery, Northwest Clinics Alkmaar, Alkmaar, The Netherlands; 2 Department of Surgery, Onze Lieve Vrouwe Gasthuis, Amsterdam, The Netherlands; 3 Department of Surgery, Utrecht University Medical Center, Utrecht, The Netherlands

**Keywords:** carotid endarterectomy, patching, patch angioplasty, primary closure, restenosis, ipsilateral stroke

## Abstract

**Objectives** Guidelines recommend routine patching to prevent restenosis following carotid endarterectomy, mainly based on studies performed many years ago with different perioperative care and medical treatment compared with current standards. Aim of the present study was to compare primary closure (PRC) versus patch closure (PAC) in a contemporary cohort of patients.

**Methods** Consecutive patients treated by carotid endarterectomy for symptomatic stenosis between January 2006 and April 2016 were retrospectively analyzed. Primary outcome was restenosis at 6 weeks and 1 year and occurrence of ipsilateral stroke. Secondary outcomes were mortality, complications, and reintervention rates.

**Results** Five hundred carotid artery endarterectomies were performed. Fifty-nine patients were excluded because eversion endarterectomy was performed or because they were asymptomatic. PRC was performed in 349 and PAC in 92 patients. Restenosis at 6 weeks was 6.0% in the PAC group versus 3.0% in the PRC group ( *p* = 0.200). Restenosis at 1 year was 31.6 versus 14.1%, respectively ( *p* = 0.104). No difference was found for stroke (3.4 vs 1.1%, *p* = 0.319), death (1.1 vs 0.0%, *p* = 0.584), or other complications (1.1 vs 0.0%, *p* = 0.584), respectively.

**Conclusions** It remains unclear whether routine patching should be recommended for all patients. A strategy of selective patching compared with routine patching, based on internal carotid artery diameter and other patient characteristics, deserves further investigation.

Carotid endarterectomy is effective in preventing recurrent stroke in patients with symptomatic carotid artery stenosis. 1 2 Low postoperative stroke and mortality rates below 3% are achieved nowadays. 3

One of the debated aspects of carotid endarterectomy technique is the type of closure applied. Restenosis rates of 1 to 36% after primary closure (PRC) implied the need for alternative techniques to reduce these rates. 4 5 6 Many guidelines recommend routine patching for most patients to prevent restenosis, mainly based on similar findings as Rerkasem and Rothwell published in a systematic review and meta-analysis in 2009. 7 8 They found a significant reduction in restenosis and ipsilateral stroke following patch angioplasty as compared with PRC. 7 8 However, the quality of included trials was generally poor and the studies were performed over 20 years ago. It is clear that current best medical treatment and stroke risks have significantly improved 3 and data from those trials might not be applicable to current medical practice anymore. Moreover, none of the included studies investigated a strategy of selective patching. Maertens et al found no difference in 30-day stroke and death rates between PRC and patch angioplasty when performing PRC in all patients with an internal carotid artery diameter of 5 mm or more. 9 Although patch angioplasty could reduce restenosis rates, it also increases bleeding risk, carotid occlusion time, procedure time, thrombus formation and carries the risk of infection and pseudoaneurysm formation. 9

Therefore, uncertainty remains about the exact role of routine patching, PRC, and selective patching. Objective of this study was to report the rates of restenosis, postoperative stroke, or transient ischemic attack and complications following carotid endarterectomy and compare these outcomes between patients where PRC and patch closure (PAC) after carotid endarterectomy were performed.

## Methods

This study was approved by the Noordwest Clinics Alkmaar Ethics Committee and the requirement for informed consent was waived by the committee.

### Patient Selection

All consecutive patients treated by carotid endarterectomy for symptomatic carotid artery stenosis in the Northwest Clinics between January 2006 and December 2016 were included in this study. A symptomatic carotid artery stenosis was defined as an internal carotid artery stenosis with an ipsilateral stroke, transient ischemic attack, or amaurosis fugax within 6 months before presentation. Carotid endarterectomy was performed in patient with a symptomatic stenosis of 50 to 99% (males) or 70 to 99% (females). Six dedicated vascular surgeons with broad experience with carotid endarterectomy operated all patients.

Patients operated after previous ipsilateral carotid endarterectomy or for reasons other than atherosclerotic stenosis (such as traumatic vascular injury or dissections) were excluded.

### Preoperative Care and Diagnostic Workup

All patients were evaluated by a neurologist for the diagnosis of transient ischemic attack, stroke, or amaurosis fugax. Duplex ultrasound was performed to identify internal carotid artery stenosis and to determine degree of stenosis according to the North American Symptomatic Carotid Endarterectomy Trial criteria. 10

Secondary prevention in the form of a daily dose of 75 mg clopidogrel and 40 mg simvastatin was immediately started since January 2014 and continued during and after carotid endarterectomy. Before 2014, a combination of acetylsalicylic acid (80mg, once a day) and dipyridamole (200 mg, twice a day) was used as standard thrombocyte aggregation inhibitor therapy instead of clopidogrel. A computed tomography angiography scan was performed for preoperative planning and confirmation of duplex findings. Blood pressure and diabetes management was instituted when indicated. Patients were scheduled for carotid endarterectomy as soon as possible and at least within 2 weeks after presentation.

### Surgical Technique

Carotid endarterectomy was performed under general anesthesia. For monitoring electro-encephalography (EEG) was used and shunting was only performed if indicated by EEG abnormalities occurring after arterial clamping. Carotid endarterectomy was performed as previously described 11 with attention to the following aspects: (1) a no-touch technique was applied; (2) heparin (5,000 international units) was administered before clamping; (3) Kunlin sutures to fixate the intima were only placed when indicated by a loose intimal flap; (4) adequate flushing from the common, internal, and external carotid arteries followed by rinsing with heparin–saline solution before completing closure of the arteriotomy; (5) releasing flow to the external carotid artery first followed after five heartbeats by the internal carotid artery. No routine placement of wound drains was performed. Three surgeons used PAC only in internal carotid arteries with a diameter of < 5 mm (selective patching), while the other three surgeons applied routine PAC. When PAC was applied, a dacron patch was used.

After completion of the operation, patients were transferred to the recovery unit for postoperative monitoring. Maximum systolic blood pressure thresholds were applied based on baseline systolic blood pressure and were usually 150 to 160 mm Hg. When systolic blood pressures were stable below the threshold for at least 4 hours, the patient was transferred to the ward.

### Data Collection and Follow-Up

Patient demographics, baseline risk factors, carotid duplex studies, indications, intraprocedural data, closure technique, periprocedural complications, and long-term outcomes were retrospectively collected. Data were derived from electronic medical records, clinical records, and imaging reports. All patients had a postoperative duplex ultrasound and a visit to the outpatient clinic 6 weeks after the procedure. The primary outcome measures were restenosis at 6 weeks and 1 year and the occurrence of ipsilateral stroke. Secondary outcome measures were death, incidence of perioperative complications, and reintervention rates.

### Statistical Analysis

Statistical analyses were performed using SPSS 20 software (IBM Corp, Armonk, NY). Fisher's Exact test was used to compare categorical variables and one-way analysis of variance was used for continuous variables. A two-tailed probability value of *p* < 0.05 was considered significant. Values are presented as mean ± standard deviation or number (%), unless stated otherwise.

## Results

### Patient Characteristics and Follow-Up

Between January 2006 and December 2016, a total of 468 patients underwent 500 carotid artery endarterectomies for internal carotid artery stenosis. Of these, 23 were performed in asymptomatic patients and 36 were treated by eversion endarterectomy and were excluded for further analysis. The remaining endarterectomies ( *n* = 441) were performed in symptomatic internal carotid artery stenosis. PRC was performed in 79.1% ( *n* = 349) and PAC in 20.9% ( *n* = 92). Patient characteristics are summarized in Table 1 and were divided according to the type of closure that was applied following endarterectomy. There were more patients with hyperlipidemia in the PRC group (49.5%) compared with the PAC group (37.9%, *p* = 0.070) but there was no difference in use of statins. A significantly smaller proportion of patients in the PRC group used clopidogrel (19.4%) when compared with the PAC group (56.5%, *p* < 0.000). For all other baseline characteristics, there were no significant differences between the study groups.

**Table 1:** Baseline characteristics

	PRC ( *n* = 349)	PAC ( *n* = 92)	*p* Value a
Age, years, mean (SD)	70.41 (9,118)	69.42 (9,199)	0.356 b
Male gender (%)	244 (69,9)	60 (65,2)	.379
Smoking (current) (%)	103 (42,6)	32 (45,1)	0.785
Smoking (ever) (%)	203 (95,3)	57 (96,6)	1.000
Hyperlipidemia (%)	163 (49,5)	33 (37,9)	0.070
Diabetes (%)	77 (22,2)	14 (15,2)	0.151
CAD (%)	91 (26,2)	24 (26,1)	1.000
COPD (%)	19 (5,5)	9 (9,8)	0.150
GFR < 60 (%)	85 (25,1)	22 (24,7)	1.000
Contralateral CEA (%)	149 (43,3)	40 (44,0)	1.000
Statin use (%)	167 (50,6)	44 (48,4)	0.724
Clopidogrel (%)	67 (19,4)	52 (56,5)	0.000
Complication (%)	39 (11,2)	11 (12,0)	0.854

Abbreviations: CAD, coronary artery disease; CEA, carotid endarterectomy; COPD, chronic obstructive pulmonary disease; GFR, glomerular filtration rate; PRC, primary closure; PAC, patch closure; SD, standard deviation.

a Fisher's exact test (exact sig. 2-sided).

b One-way analysis of variance.

The median follow-up was 6 weeks (range: 0–130 months). According to the local protocol, all patients were scheduled for a follow-up visit and duplex ultrasound 6 weeks following carotid endarterectomy. Twenty patients in the PRC group (5.7%) and eight in the PAC group (8.7%) were lost to follow-up at 6 weeks. Four patients in the PRC group died. The remaining patients were lost to follow-up for unknown reasons.

Follow-up data at 1 year was available for 85 patients in the PRC group and 19 in the PAC group. Reasons for the follow-up visits beyond the 6 weeks according to the local protocol were contralateral stenosis, patient preference, or individual surgeon's preference. None of the patients returned with an ipsilateral symptomatic restenosis or stroke.

### Outcome Measures

Overall, there was no statistically significant difference in the number of complications between the PRC and PAC groups (11.2 vs 12.0%, *p* = 0.854). There were more reinterventions for postoperative bleeding in the PAC group (10.9 vs 5.4%), although this difference was not statistically significant ( *p* = 0.094). No statistically significant differences in stroke, death, and other complications were found (see Table 2 ). Four patients died, all in the primary closed group. Causes of death were hypertensive intracranial bleeding ( *n* = 2) and myocardial infarction ( *n* = 2). Another four patients, all in the PRC group developed complications other than restenosis, stroke, or death. These complications were nerve injury ( *n* = 1), hyperperfusion syndrome ( *n* = 1), hypotension and wound infection ( *n* = 1, both in the same patient), and internal carotid artery occlusion ( *n* = 1), which required reoperation and thrombectomy.

**Table 2:** Thirty-day complication rates

	PRC ( *n* = 349)	PAC ( *n* = 92)	*p* Value a
Reintervention for bleeding (%)	19 (5,4)	10 (10,9)	0.094
Stroke (%)	12 (3,4)	1 (1,1)	0.319
Death (%)	4 (1,1)	0 (0,0)	0.584
Other (%)	4 (1,1)	0 (0,0)	0.584
None (%)	310 (88,8)	81 (88,0)	0.854

Abbreviations: PRC, primary closure; PAC, patch closure.

a Fisher's exact test (exact sig. 2-sided).

At the duplex scan, 6 weeks after carotid endarterectomy 10 out of 329 (3.0%) patients in the PRC and 5 out of 84 (6.0%) patients in the PAC group had a significant (i.e., > 50%) restenosis. The difference was not statistically significant. At 1 year follow-up, a duplex scan was available for 85 out of 329 (25.8%) patients in the PRC group and 19 out of 84 (22.6%) patients in the PAC group. No statistically significant difference could be found in the proportion of patients with a restenosis ( Table 3 ). In Table 4 , the degree of stenosis found at 6 weeks and 1 year follow-up is summarized for < 50%, 50 to 70%, and > 70% stenosis, respectively. A higher proportion of patients in the PRC group (313 out of 329; 95.1%) had no restenosis at all, compared with 75 out of 84 (89.3%) patients in the PAC group. After 1 year, the difference in the proportion of patients without any restenosis between groups increased, although no statistical significance could be reached.

**Table 3:** Restenosis after 6 weeks and 1 year

	PRC	PAC	*p* Value a
Restenosis b 6 weeks (%)	*n* = 329 10 (3,0)	*n* = 84 5 (6,0)	0.200
Restenosis b 1 year (%)	*n* = 85 12 (14,1)	*n* = 19 6 (31,6)	0.092

Abbreviations: PRC, primary closure; PAC, patch closure.

a Fisher's exact test (exact sig. 2-sided).

b Significant (> 50%) restenosis according to the NASCET (North American Symptomatic Carotid Endarterectomy Trial) criteria.

**Table 4:** Degree of restenosis after 6 weeks and 1 year

	PRC	PAC	*p* Value a
Restenosis 6 weeks (%)	*n* = 329	*n* = 84	
< 50%	6 (1,8)	4 (4,8)	0.124
50–70%	5 (1,5)	2 (2,4)	0.634
> 70%	5 (1,5)	3 (3,6)	0.209
None	313 (95,1)	75 (89,3)	0.068
Restenosis 1 year (%)	*N* = 85	*N* = 19	
< 50%	6 (7,1)	2 (10,5)	0.636
50–70%	7 (8,2)	3 (15,8)	0.385
> 70%	5 (5,9)	3 (15,8)	0.159
None	67 (78,8)	11 (57,9)	0.078

Abbreviations: PRC, primary closure; PAC, patch closure.

a Fisher's exact test (exact sig. 2-sided).

## Discussion

The present study compared the rate of restenosis, stroke, reinterventions, and other complications between PRC and PAC in a large contemporary cohort of patients that underwent carotid endarterectomy for internal carotid artery stenosis. During duplex follow-up at 6 weeks, no statistically significant difference was found in the proportion of patients with a significant (> 50%) restenosis between groups. The subgroup of patients with a follow-up duplex scan after 1 year was too small and the risk for selection bias in this group was considered too high to draw any conclusions. The postoperative stroke rate was low in both the PRC and PAC group (3.4 vs 1.1%, respectively) and not statistically different.

These findings are conflicting with those described in the systematic review by Rerkasem and Rothwell from 2009, 7 where a significant reduction in stroke, ipsilateral stroke, and restenosis was found following PAC. However, trials included in this review were published 11 to 30 years ago (1987–2006) in a time where perioperative care and medication were different compared with current standards. Moreover, the sample sizes were relatively small, data were not available from all trials, and there was significant loss to follow-up. In a more recently published systematic review and meta-analysis by the same authors, three more trials were included in the analysis and two recent trials reported nonsignificant trends toward an increased operative risk of stroke and death with PAC, further increasing doubt in the currently available evidence supporting the routine use of PAC. 12

Added to that, Malas et al performed a post-hoc analysis of 1,151 patients included in the carotid endarterectomy arm of the CREST (Carotid Revascularization Endarterectomy versus Stenting Trial) trial and compared those that underwent PRC with PAC. 13 Although they found a significant reduction in restenosis in the PAC group at 2 years, there was no difference in perioperative stroke or 4-year ipsilateral stroke. 13 Futhermore, the NSQIP (National Surgical Quality Improvement Program) data analysis of 3,845 patients demonstrated that in contrast to other parameters the technical aspects of carotid endarterectomy were not predictive for postoperative stroke or death. 14

The most recent paper and largest cohort study by Avgerinos et al reported results of a retrospective cohort of 1,737 carotid endarterectomies treated between 2000 and 2010 with a median follow-up of 49 months. 15 They found no significant differences in the rate of restenosis, ipsilateral stroke, or death between PRC of PAC. On multivariate and cox-regression analysis, the type of closure had no predictive value for restenosis or perioperative and long-term outcomes. The only predictive factors were the presence of symptomatic stenosis, heart failure or renal failure, and the use of statins. 15

All these recent findings indicate that the recommendations for routine use of PAC might be unjustified. The trials on which these recommendations were based are outdated and performed in an era with less effective secondary prevention and higher perioperative stroke risks. With the declining perioperative stroke rates and the recent findings described above, it seems that the impact of closure technique on carotid endarterectomy outcomes is overestimated. Contemporary trials such as the CREST trials demonstrated a restenosis rate of 3 to 7% within 2 to 4 years. 5 13 A small minority of these are symptomatic and < 0.5% of the overall group of patients that undergo carotid endarterectomy develop a symptomatic restenosis. This further questions the clinical significance of the rate of restenosis.

There are some limitations to the conclusions that can be drawn from this study that warrant further discussion. First, due to the retrospective nature of the study selection bias cannot be ruled out and the influence of unknown confounders is hard to estimate. Although the sample size is reasonable, the relatively low number of postoperative events might render the study underpowered to detect some possible relevant differences, especially for the small subgroup of patients that had data available for a follow-up of 1 year.

The reason to use PAC or PRC in this cohort was impossible to obtain from the available data. The most important reason to use a patch in our hospital for those surgeons applying selective patching is an internal carotid artery diameter of < 5 mm. Thus, smaller internal carotid arteries are more likely to be closed using a patch but these arteries might also be more prone to (re)stenosis. This could be a source of bias leading to a relative overestimation of the rate of restenosis in the PAC group. Additionally, a larger part of the cohort was operated by surgeons that preferred selective patching which is another source of selection bias. Therefore, based on the data presented in this paper, we cannot determine the role of selective patching.

In the PRC group, a significantly smaller proportion of patients used clopidogrel. This can be explained by the fact that in the earlier cohort acetylsalicylic acid and dipyridamole were standard therapy and since 2014 this was replaced by clopidogrel. In the later cohort more surgeons had adopted the routine use of PAC. These two developments caused the higher proportion of patients receiving clopidogrel in the PAC group. Although the use of clopidogrel might have influenced the rate of perioperative stroke, this would most likely cause an overestimation of the stroke rate in the PRC group. If it would be possible to correct for this, it would lead to a decrease in the rate of strokes in the PRC groups and further confirm our findings that there is no increased stroke risk in PRC compared with PAC. The higher proportion of patients using clopidogrel in the PAC group might be a confounder that leads to the higher rate of postoperative bleeding in the PAC group.

Most patients in our study had a relatively short follow-up of 6 weeks which is insufficient to draw any conclusions on long-term outcomes. However, on physical grounds, the effect of using a patch or not for closure is a direct effect. The patch is used to negate the effect of the bites of the sutures into the vessel wall and this effect can reliably be measured by duplex scanning even on short-term follow-up.

Moreover, the study of Avgerinos et al 15 showed no difference in 5- or 10-year follow-up in restenosis and most restenosis occurred in the first year. Interestingly, a very recent study on the hemodynamics of carotid endarterectomy closure techniques found no favorable flow dynamics after patching because incorporation of a patch increases areas of low wall shear stress and high oscillatory shear index at the bifurcation. 16

Finally, all postoperative deaths ( *n* = 4) were in the PRC group. However, when further looking into the causes of death (myocardial infarction, intracranial bleeding and palliative care after epilepsy and pneumonia), we were unable to relate these causes to the type of closure applied.

## Conclusions

Although firm conclusions cannot be drawn on the basis of this retrospective study, we confirmed the findings of previous recent studies that indicate that strong recommendations for routine use of PAC might be unjustified. Since the recommendation for routine patching is based on outdated trials of questionable methodological quality, there is room for a high-quality randomized controlled trial comparing PRC and PAC in a contemporary cohort of patients that require carotid endarterectomy. Ideally patients would be stratified according to internal carotid artery diameter to explore whether an approach of selective patching is the most beneficial. Although it seems evident that never using a patch should not be recommended, we doubt that patching is beneficial in all cases.
